# 45° helical plates are a valid alternative to straight plates for treatment of proximal humeral shaft fractures

**DOI:** 10.1002/jor.26020

**Published:** 2024-11-25

**Authors:** Tatjana Pastor, Ivan Zderic, Frank J. P. Beeres, Nader Helmy, R. Geoff Richards, Philipp Kriechling, Ludmil Drenchev, Hristo K. Skulev, Boyko Gueorguiev, Torsten Pastor

**Affiliations:** ^1^ AO Research Institute Davos Davos Switzerland; ^2^ Department of Orthopaedics and Traumatology Bürgerspital Solothurn Solothurn Switzerland; ^3^ Department of Orthopaedic and Trauma Surgery Lucerne Cantonal Hospital Lucerne Switzerland; ^4^ Department of Health Science and Medicine University of Lucerne Luzern Switzerland; ^5^ Department of Orthopedics Balgrist University Hospital Zurich Switzerland; ^6^ Bulgarian Academy of Sciences, Institute of Metal Science “Acad. A. Balevski” Sofia Bulgaria; ^7^ Medical Faculty, University of Zurich Zurich Switzerland

**Keywords:** biomechanics, helical plate, MIPO, proximal humeral shaft fracture

## Abstract

Helical plates used for proximal humeral shaft fracture fixation avoid the radial nerve distally as compared to straight plates. To investigate in a human cadaveric model the biomechanical competence of straight lateral plates versus 45° helical plates used for fixation of proximal comminuted humeral shaft fractures, eight pairs of human cadaveric humeri were instrumented using either a long straight PHILOS plate (Group 1) or a 45° helical plate (Group 2) for treatment of an unstable proximal humeral shaft fracture. All specimens were tested under non‐destructive quasi‐static loading in axial compression, internal and external rotation, and bending in four directions. Subsequently, progressively increasing cyclic loading in internal rotation was applied until failure and interfragmentary movements were monitored by motion tracking. Axial displacement (mm) was 3.13 ± 0.31 in Group 1 and 2.60 ± 0.42 in Group 2, *p* = 0.015. Flexion/extension deformation (°) in Group 1 and Group 2 was 0.56 ± 0.42 and 0.43 ± 0.23, *p* = 0.551. Varus/valgus deformation (°) was 6.39 ± 0.44 in Group 1 and 5.13 ± 0.87 in Group 2, *p* = 0.012. Shear (mm) and torsional (°) displacement were 5.36 ± 0.76 and 17.75 ± 1.06 in Group 1, and 5.03 ± 0.46 and 16.79 ± 1.36 in Group 2, *p* ≥ 0.090. Cycles to catastrophic failure were 10000 ± 1401 in Group 1 and 9082 ± 1933 in Group 2, *p* = 0.708. From a biomechanical perspective, 45° helical plating is associated with lower axial and varus/valgus displacement under axial loading and demonstrates comparable resistance to failure versus straight plating. Therefore, 45° helical plates can be considered as a valid alternative to straight plates for treatment of proximal humeral shaft fractures.

## 1 INTRODUCTION

Proximal humeral fractures constitute approximately 4%–5% of all human fractures.^1^ In dislocated or complex humeral shaft fractures surgery is generally preferred.^2^ Surgical treatment options include plate and nail osteosynthesis that both have been investigated previously.^2^, ^3^, ^4^, ^5^ A possible downside of the nailing is the potential injury to the rotator cuff, which might not be desirable and plate osteosynthesis might be chosen in younger patients by the treating surgeon. The use of minimally invasive plate osteosynthesis (MIPO) already led to lower nerve injury rates and less soft tissue damage—as compared to the conventional open reduction and internal fixation technique (ORIF).^6^, ^7^, ^8^, ^9^ However, the use of a straight plate technique endangers the radial nerve distally, as it is in close proximity to the inserted plate.^10^ Helical plates can wrap around the bone in the anterior direction to avoid the radial nerve distally and several reports demonstrate good clinical results using this technique in proximal humeral shaft fractures.^11^, ^12^, ^13^, ^14^, ^15^, ^16^, ^17^ The best helical plate design, however, is not universally agreed upon in the current literature as there are existing reports related to helical implants twisted in the range from 45° to 90°.^11^, ^12^, ^13^, ^14^, ^15^, ^16^, ^17^ Recently, a biomechanical investigation discovered greater fracture gap shear displacements and an inferior biomechanical competence in 90° helical plate designs as compared to straight plates.^18^ Furthermore, 45° helical plates are more suitable from an anatomic point of view when inserted with the MIPO technique, as they are pushed through the weaker middle part of the deltoid insertion, which is not possible with 90° helical plates.^10^ Therefore, the aim of this study was to investigate the biomechanical competence of long 45° helical plates versus conventional straight plates in proximal‐third comminuted humeral shaft fractures in a human cadaveric bone model. It was hypothesized that 45° helical plates demonstrate a comparable biomechanical behavior versus straight plates and may be considered as their valid alternative.

## 2 METHODS

### 2.1 Specimens and study groups

**Figure 1:**
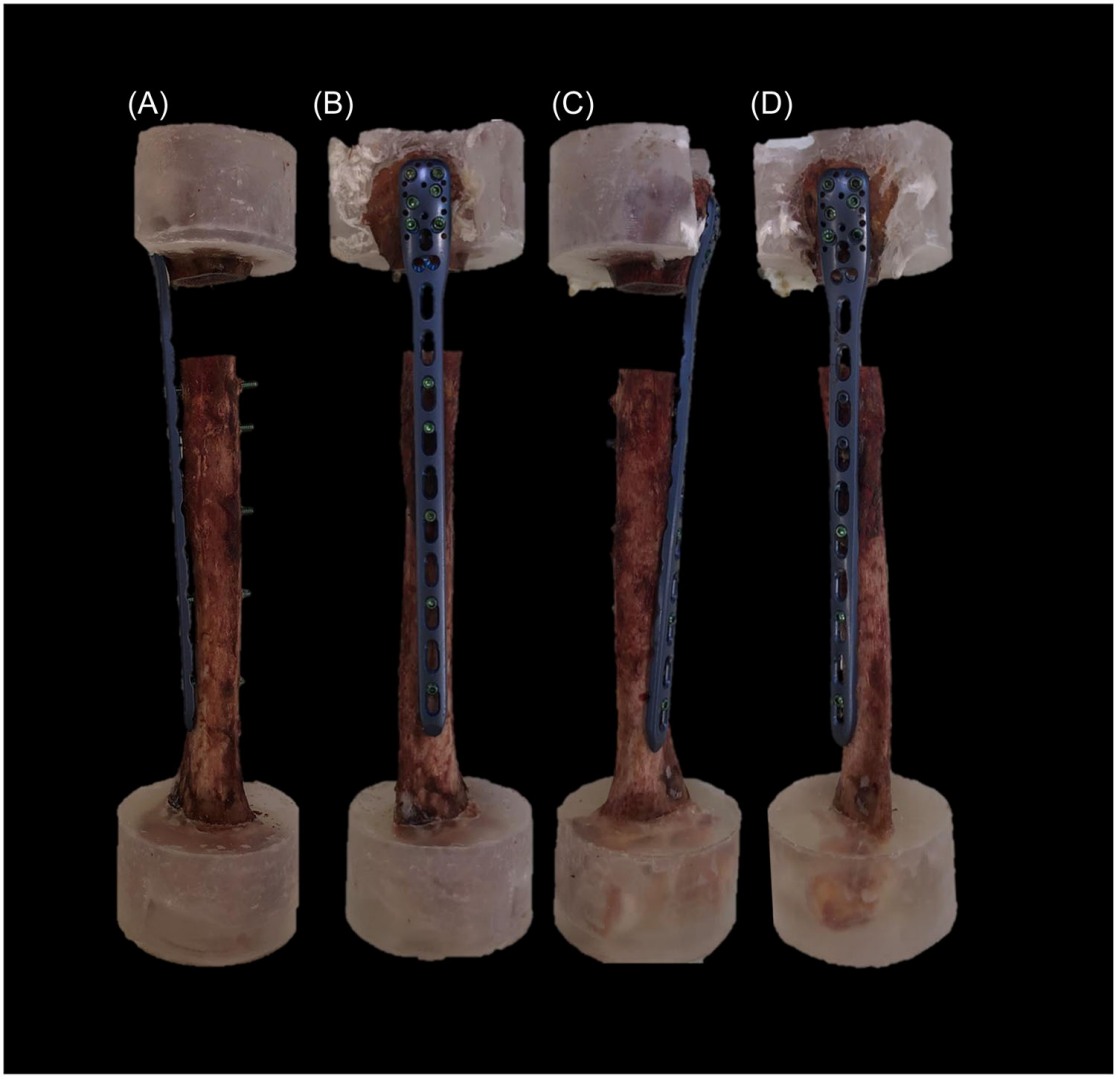
Exemplified specimens of the same donor with an osteotomy gap, simulating a humeral shaft fracture of the proximal third treated via straight (left, Group 1) or 45° helical (right, Group 2) plating in anterior (A and C) and lateral (B and D) views.

Eight pairs of fresh‐frozen (−20°C) human cadaveric humeri from five female and three male donors aged 81.6 years on average (range 69–95 years) were used. All donors gave their informed consent inherent within the donation of the anatomical gift statement during their lifetime. Specimens were pairwise assigned to two groups for plating with either a long 10‐hole straight PHILOS plate (PHILOS long, 232 mm, DePuy Synthes) mounted on the lateral side of the humerus (Group 1) or a long custom bend 10‐hole 45° helical PHILOS plate mounted from the proximal lateral to the distal anteromedial side of the humerus (Group 2), (Figure 1). The specimens were thawed at room temperature (20°C), freed from all soft tissues, and subjected to computed tomography scanning before testing at a slice thickness of 0.63 mm (Revolution EVO, GE Medical Systems AG) to calculate volumetric bone mineral density (BMD) using a phantom (European Forearm Phantom QRM‐BDC/6, QRM GmbH).^19^

### 2.2 Specimen preparation

All humeri were instrumented under fluoroscopic control (Siemens ARCADIS Varic, Siemens Medical Solutions AG) according to the implant manufacturer's guidelines. Using bending irons, the plates in Group 2 were prebent to a 45° twist before being attached to the bone, and secured with two Kirschner (K−) wires and two repositioning forceps. Pilot holes of 2.5 mm were predrilled using an angular stable drill sleeve through plate holes 1, 3, 5, 7, and 8 counting from the distal plate end in bicortical fashion. The proximal six plate holes were only predrilled to the second cortex of the humeral head aiming for a distance of 5 mm to the second cortex. This approach was chosen as the current study aimed to test the plate constructs resulting from a MIPO technique application, where it is not possible to fit all screw holes in the humeral head. Usually, only four proximal screws are inserted; however, in certain situations, two additional screws might be added without endangering the axillary nerve. Final plate securing was achieved via locking screw fixation through these pilot holes using a total of five bicortical 3.5 mm locking screws distally and a total of six monocortical 3.5 mm locking screws proximally. A 1.5 Nm torque limiter was used for screw locking. Subsequently, an AO/OTA 12 C3 fracture was simulated by means of a 3 cm osteotomy gap starting 4 cm below the greater tuberosity, standardized in all specimens by using a cutting jig. Following instrumentation, the proximal and distal 35 mm ends of the specimens were embedded in polymethylmethacrylate (PMMA, SCS‐Beracryl D28, Suter Kunststoffe AG) cylindrical forms, sparing the plates proximally. The anatomical axis—defined as a straight line connecting the center of rotation of the glenohumeral joint and the central aspect between the medial and lateral epicondyles at the elbow^20^, ^21^—was aligned with the axes of both embedding cylindric forms. Optical markers were attached to the humeral head and shaft at a distance of 5 mm to the osteotomy gap for motion tracking as used in previous work.^18^

### 2.3 Test setup

**Figure 2:**
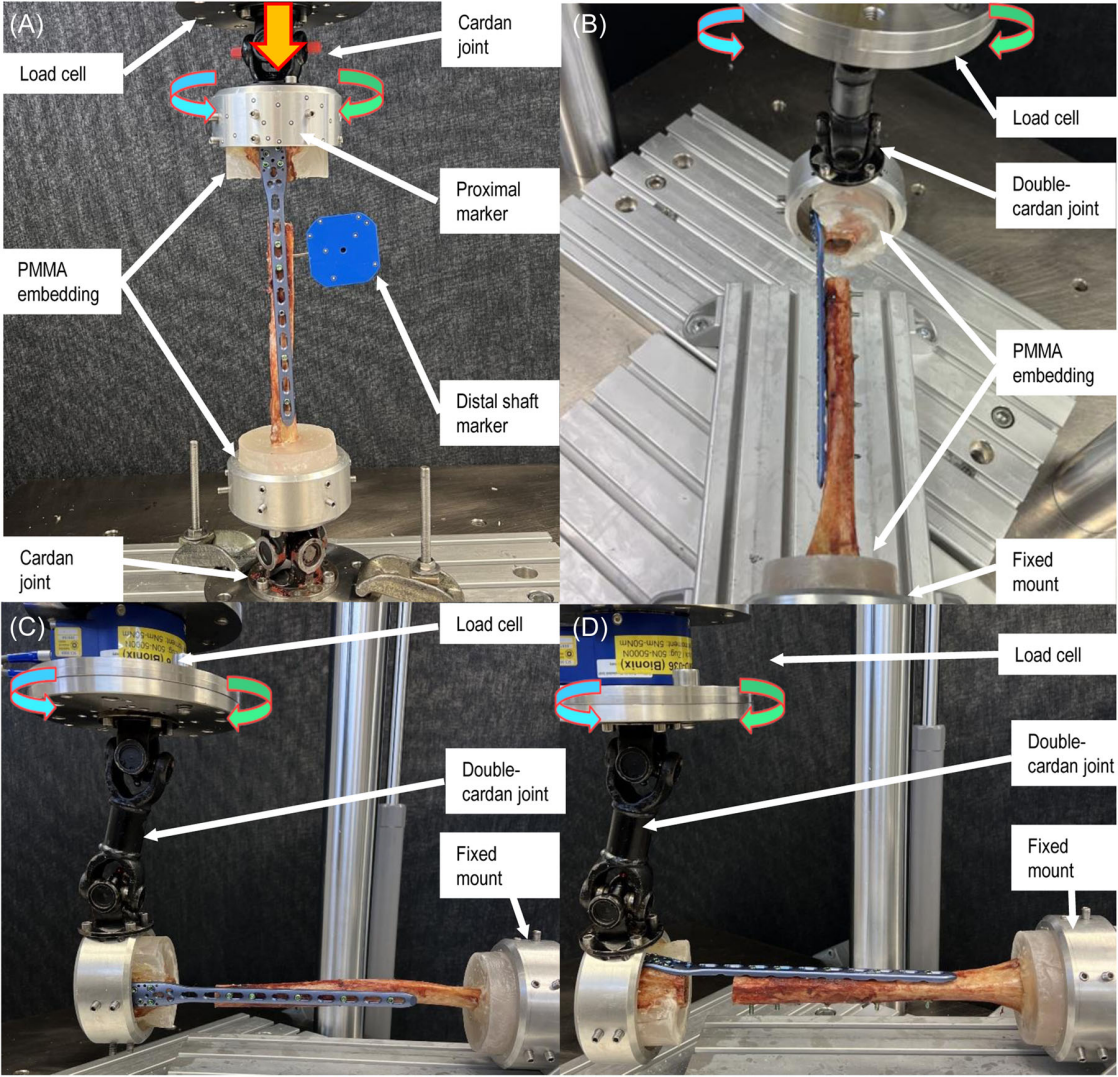
Setup with a specimen from Group 1 mounted for (A) axial compression (Test 1) and internal/external rotation tests (Test 2, 3, and 8) equipped with markers for motion tracking; (B, C) pure bending tests in the coronal plane (varus/valgus) (Tests 4–5); (D) pure bending tests in the sagittal plane (flexion/extension) (Tests 6 and 7).

Biomechanical testing was performed on a servo‐hydraulic test system (Mini Bionix II 858, MTS Systems Corp.) equipped with a 4 kN load cell in dry environment at room temperature (20°C). Test setup and loading protocol were adopted from previous studies (Figure 2 *)*.^18^, ^21^, ^22^, ^23^ Each specimen underwent seven non‐destructive quasi‐static tests followed by destructive cyclic testing. First, axial compression was applied to all specimens, which were connected between two cardan joints with the humeral mechanical axis aligned with the machine axis (Test 1). Second, torsion was applied in both internal and external rotation to simulate the forces and moments generated by the rotator cuff during internal and external rotation of the humerus (Tests 2 and 3). Third, to replicate pure bending, each specimen was mounted between a fixed basis and a double cardan joint. In this position varus and valgus pure bending was performed, simulating the loads and moments that the deltoid muscle and rotator cuff generated in the humerus during both abduction and adduction (Tests 4 and 5). Additionally, each specimen underwent pure flexion and extension bending, which replicated the flexion and extension of the elbow, by rotating it by 90°. With this test setup configuration, the axis of the humeral shaft and the cardan's rotation intersected at a 90° angle, enabling the conversion of actuator torque into pure bending moments acting on the humerus (Tests 6 and 7). During Tests 4–7, the axial force along the actuator axis was maintained at 0 N to keep the specimen free from shear stresses. Moreover, the inherent kinematic characteristics of the double cardan joint compensated potential axial stresses, while the intersection of the two mechanical axes eliminated potential torsional moments. Finally, all specimens were reorientated to align their mechanical axis with the machine axis and destructive cyclic testing in internal rotation was performed (Test 8).

### 2.4 Loading protocol

The loading protocol for axial compression (Test 1)—related to axial movement of the machine actuator—consisted of three non‐destructive quasi‐static ramps from 0 to 250 N at a rate of 0.05 mm/s, whereas the loading protocol for Tests 2–7—related to torsional movement of the machine actuator—consisted of three non‐destructive quasi‐static ramps in both directions from 0 to ±5 Nm at a rate of 0.5°/s. In all quasi‐static tests, the first two ramps allowed for specimen's settling, whereas the third ramp was used for data evaluation.

The loading protocol for destructive cyclic testing (Test 8) implemented progressively increasing sinusoidal loading in internal rotation at 1 Hz and was adopted from previous work.^21^ This approach was chosen due to the fact that the upper extremities are predominantly loaded in torsion.^24^, ^25^ The axial load was kept at 0 N during the whole cyclic test. Whereas the valley torque of each cycle was kept at a constant level of 0.5 Nm, the peak torque, starting at 5 Nm, increased at a rate of 0.002 N/cycle. Reaching an angle of 90° by the machine actuator relative to its position at the start of the test was defined as a test stop criterion.

### 2.5 Data acquisition and analysis

Machine data in terms of axial load, torque, axial displacement, and torsional angle were recorded from the machine controllers at 128 Hz. Based on these data, axial, bending, and torsional construct stiffness was calculated from the ascending slope of the load–displacement curve from the quasi‐static ramps within the linear range between 0 and 250 N for axial loading (Test 1), and within ±5 Nm for torsional loading (Tests 2 and 3) and pure bending (Tests 4–7), respectively. Further, to assess the interfragmentary movements in all six degrees of freedom, the coordinates of the markers attached to the specimens were acquired throughout Tests 1, 2, 3, and 8 at 75 Hz by means of stereographic optical measurements using contactless full‐field deformation technology (Aramis SRX, GOM GmbH).

Based on these data, interfragmentary movements in terms of axial displacement—defined as the relative translational proximal to distal humeral shaft movement along the humeral shaft axis—, varus/valgus displacement—defined as the relative angular proximal to distal humeral shaft bending movement in the coronal plane—, and flexion/extension displacement—defined as the relative angular proximal to distal humeral shaft movement in the sagittal plane—was assessed under 250 N axial compression. Shear displacement—defined as the relative translational humeral shaft movement in the fracture plane—and torsional displacement—defined as the relative angular proximal to distal humeral shaft torsional movement in the transverse plane—were assessed between −5 and +5 Nm torsional loading.

During cyclic testing, interfragmentary shear and torsional displacements were analyzed after 1000, 2000, 3000, 4000, 5000, 6000, 7000, and 8000 test cycles under valley loading to assess the degradation of construct stability over time as previously described.^26^, ^27^, ^28^, ^29^ After each destructive test the specimens were inspected visually and under fluoroscopy to evaluate their specific mode of failure. Machine data were used to detect the time point of catastrophic failure of each specimen.

### 2.6 Statistical analysis

Statistical analysis was performed with SPSS software package (IBM SPSS Statistics, V27, IBM). Shapiro–Wilk test was used to screen and prove normality of the data distribution. Significant differences with regard to outcome measures derived from quasi‐static and cyclic testing were identified with Paired‐Samples *t*‐test and General Linear Model Repeated Measures test, respectively. The level of significance was set to 0.05 for all statistical tests.

## 3 RESULTS

### 3.1 Volumetric BMD

Trabecular volumetric BMD was 131.7 ± 32.1 mg HA/cm^3^ in Group 1, and 120.9 ± 28.6 mg HA/cm^3^ in Group 2, with no significant differences between the groups (*p* = 0.333).

### 3.2 Non‐destructive quasi‐static tests

Values of the parameters of interest during the non‐destructive quasi‐static tests are presented in Table 1. Axial stiffness—calculated from Test 1—demonstrated no significant differences between the groups (*p* = 0.067). No significant differences were detected between the groups in terms of stiffness in internal and external rotation—calculated from Tests 2 and 3—(*p* ≥ 0.104). In addition, stiffness in varus and valgus—calculated from Tests 4 and 5—as well as stiffness in flexion and extension—calculated from Tests 6 and 7—showed no significant differences between the groups (*p* ≥ 0.233).

**Table 1:** Parameters of interest in each group presented in terms of mean value and standard deviation, together with corresponding *p* values from the statistical comparison between the groups.

Parameter of interest	Group 1 (straight plate)	Group 2 (helical plate)	*p* Value
Stiffness	
Axial (Nm/mm)	91.3 ± 9.6	139.1 ± 55.6	0.067
Flexion (Nm/°)	2.25 ± 0.76	2.12 ± 0.84	0.698
Extension (Nm/°)	2.23 ± 0.80	2.42 ± 1.02	0.710
Valgus (Nm/°)	0.80 ± 0.14	0.79 ± 0.19	0.891
Varus (Nm/°)	0.78 ± 0.15	1.21 ± 0.82	0.233
Internal rotation (Nm/°)	0.52 ± 0.05	0.56 ± 0.08	0.104
External rotation (Nm/°)	0.53 ± 0.04	0.53 ± 0.06	0.581
Displacement during non‐destructive testing			
Axial (mm)	3.13 ± 0.31	2.60 ± 0.42	0.015
Flexion/extension (°)	0.56 ± 0.42	0.43 ± 0.23	0.551
Varus/valgus (°)	6.39 ± 0.44	5.13 ± 0.87	0.012
Shear (mm)	5.36 ± 0.76	5.03 ± 0.46	0.124
Torsion (°)	17.75 ± 1.06	16.79 ± 1.36	0.090
Torsional displacement during cyclic testing (°)			
1000 cycles	1.35 ± 0.24	1.43 ± 0.22	0.281
2000 cycles	2.98 ± 0.27	3.05 ± 0.48	0.644
3000 cycles	4.81 ± 0.43	5.00 ± 0.99	0.505
4000 cycles	7.01 ± 0.84	7.20 ± 1.47	0.661
5000 cycles	9.63 ± 2.03	10.04 ± 2.28	0.555
6000 cycles	12.63 ± 4.07	14.04 ± 4.21	0.553
7000 cycles	17.58 ± 6.96	19.44 ± 7.25	0.573
8000 cycles	21.61 ± 7.89	24.18 ± 10.93	0.241
Shear displacement during cyclic testing (mm)			
1000 cycles	0.22 ± 0.12	0.13 ± 0.15	0.178
2000 cycles	0.53 ± 0.26	0.31 ± 0.33	0.171
3000 cycles	1.00 ± 0.49	0.55 ± 0.55	0.152
4000 cycles	1.62 ± 0.80	0.91 ± 0.86	0.121
5000 cycles	2.49 ± 1.43	1.57 ± 1.14	0.171
6000 cycles	3.64 ± 2.30	2.82 ± 2.50	0.374
7000 cycles	5.70 ± 4.43	4.79 ± 4.12	0.473
8000 cycles	8.03 ± 5.99	5.93 ± 6.00	0.831
Catastrophic failure			
Cycles to failure	10000 ± 1401	9082 ± 1933	0.708
Torque at failure (Nm)	16.78 ± 2.16	15.43 ± 2.09	0.117

Axial displacement—calculated from Test 1—was significantly higher in Group 1 compared to Group 2 (*p* = 0.015). Torsional and shear displacement—calculated from Tests 2 and 3—remained non‐significantly different between the groups (*p* ≥ 0.090). Varus/valgus displacement—calculated from Tests 4 and 5—was significantly higher in Group 1 compared to Group 2 (*p* = 0.012). In contrast, flexion/extension displacement—calculated from Tests 6 and 7—remained non‐significantly different between the groups (*p* = 0.551).

### 3.3 Destructive cyclic test

**Figure 3:**
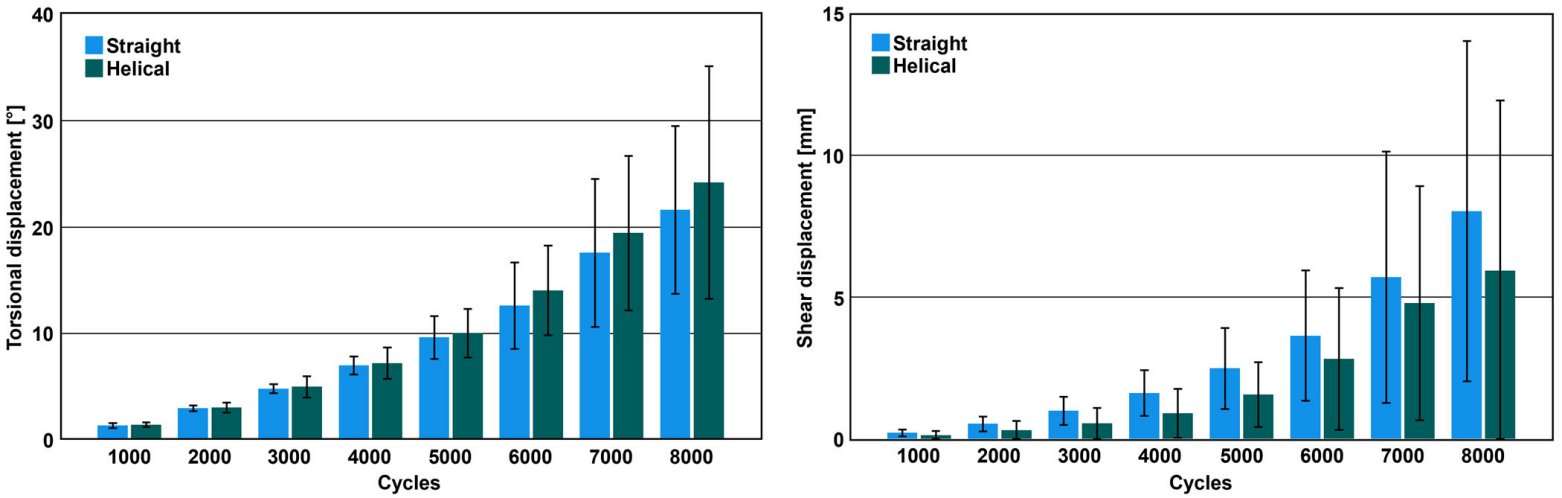
Torsional (left) and shear (right) displacement in Group 1 (Straight) and Group 2 (Helical) over the course of selected cycles (Test 8) presented in terms of mean value and standard deviation.

Values of the parameters of interest during the destructive testing—calculated from Test 8—are summarized in Table 1 and visualized in Figure 3. Shear and torsional displacement over the preselected cycles were not significantly different between the groups (*p* ≥ 0.121). Torsional displacement increased significantly over the course of preselected cycles in both groups (*p* < 0.001). Shear displacement in Group 1 increased significantly over the course of preselected cycles (*p* = 0.013). In contrast, the increase of shear displacement in Group 2 was not significantly different over the course of preselected cycles (*p* = 0.094).

### 3.4 Catastrophic failure

Cycles to catastrophic failure and the corresponding torque at failure are presented in Table 1. No significant differences were detected between the groups with regard to these two parameters of interest (*p* ≥ 0.117).

### 3.5 Failure modes

**Figure 4:**
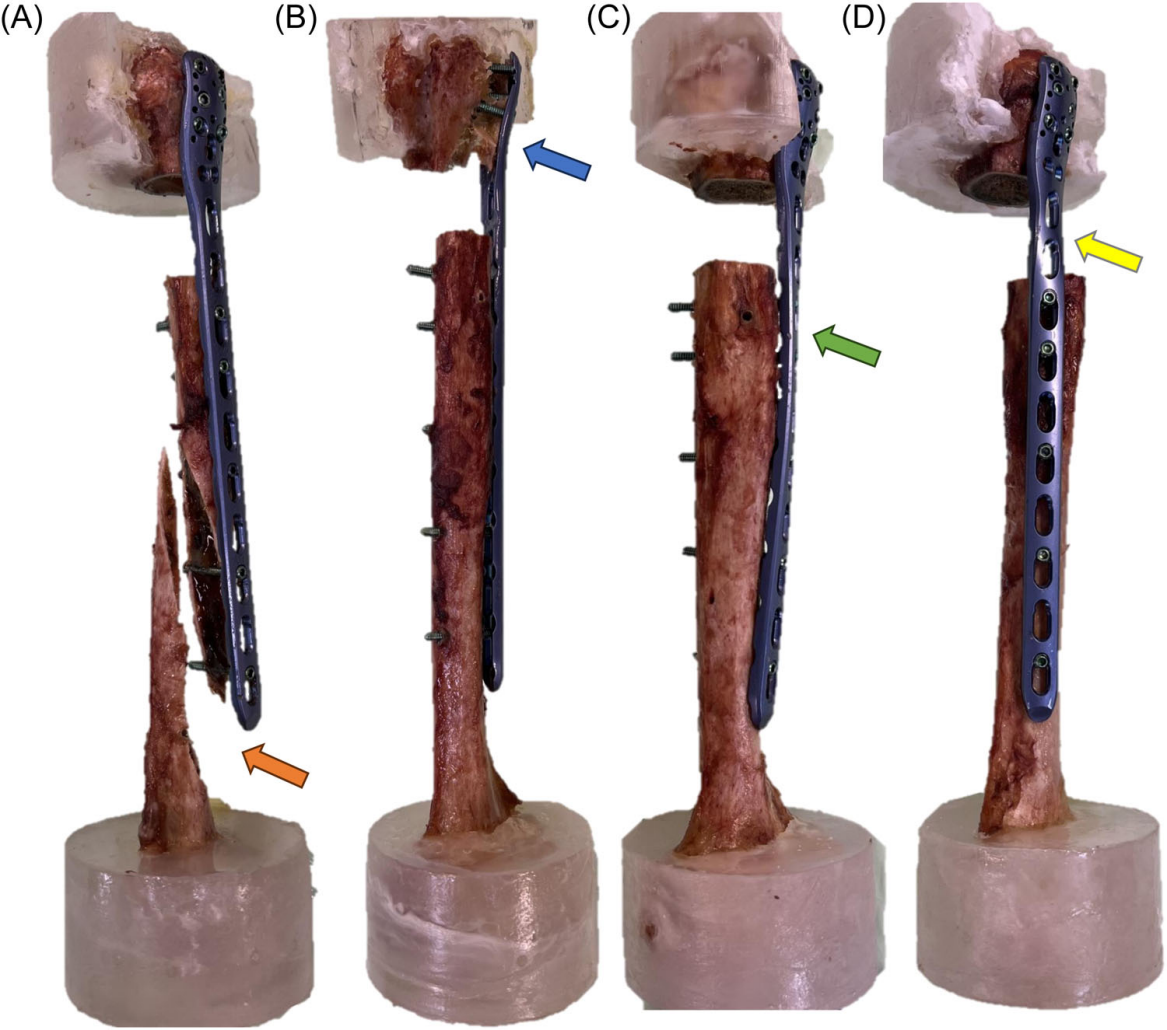
Most frequent catastrophic failure modes in both groups. (A) Anterior view of a left humerus with a straight lateral plate. Arrow indicates a spiral humeral shaft fracture. (B) The anterior view of a left humerus with a straight lateral plate. Arrow indicates anterior cut out of all six screws through the humeral head. (C) Anterior view of a left humerus with a 45° helical plate. Arrow indicates screw breakage distally to the osteotomy gap. (D) Lateral view of a left humerus with a straight plate. Arrow indicates a deformation of the plate at the osteotomy level.

The main catastrophic failure mode in both groups was the anterior screw cut out of the humeral head in five specimens of each group. Furthermore, torsional plate deformation occurred in four specimens of each group. Besides, two spiral fractures of the distal humerus were observed in Group 1 and one in Group 2. Additional screw breakage distally to the osteotomy gap occurred in two specimens of each group (Figure 4).

## 4 DISCUSSION

The current study compared the biomechanical competence of long 45° helical plates versus conventional straight plates in proximal third comminuted humeral shaft fractures. Although there are several promising clinical reports on helical plates in the literature, the optimal design is still debated. Furthermore, an alternative to the pure helical design was recently introduced. The anterolateral plating system (ALPS, Zimmer Biomet) is a 45° twisted plate with an additional anterior kink to avoid the deltoid insertion.

From an anatomic perspective, several helical plate designs with twisting from 45° to 90° as well as the ALPS design as provided by Zimmer Biomed, are suitable for ORIF as they demonstrated safe feasibility and greater distances to the radial nerve as compared to straight plates.^10^ Furthermore, Dauwe et al. reported bigger stretching of the axillary nerve during MIPO of proximal humeral shaft fractures with straight plates compared to helical plates.^30^ In addition, 6%–7% iatrogenic radial nerve damage rates were reported for MIPO with straight plates.^31^, ^32^ These promising anatomic reports are underlined with positive clinical reports for 45° and 90° helical plates and ALPS.^8^, ^9^, ^11^, ^12^, ^14^, ^15^, ^16^, ^17^, ^33^, ^34^ However, MIPO is not possible without releasing the strong anterior part of the deltoid insertion when 90° helical plates or ALPS are used.^10^ Additionally, an anatomical investigation using 10 human cadaveric humeri examined the safety of 90° helical plates using the MIPO technique and revealed the musculocutaneous nerve as being the primary structure at risk.^35^ Similar results were found for ALPS.^36^ In contrast, the 45° helical plate is pushed through the weaker middle part of the deltoid insertion and revealed greater distances to the musculocutaneous nerve as compared to both 90° helical plates and ALPS, and seems therefore more suitable for MIPO.^10^, ^36^ However, the clinical impact of a deltoid insertion release is controversy debated in the current literature.^13^, ^37^

From a biomechanical point of view, no studies have been conducted with a focus on ALPS so far. In contrast, helical plates have been investigated via a finite element analysis and higher resistance to torsional forces of 90° and 180° helical plates have been reported by Krishna et al. in comparison to straight plates.^38^ Additionally, two biomechanical studies have been published on helical plates used for proximal humeral shaft fixation so far. Straight plates, 45° helical plates, and 90° helical plates have been compared in an artificial bone model using a non‐destructive quasi‐static test setup.^23^ It was concluded that 90° helical plates were associated with higher fracture gap movements in the sagittal plane (flexion/extension); however, they demonstrated improved resistance against displacements in the coronal plane (varus/valgus) compared to straight plates during pure bending. In contrast, 45° helical plates demonstrated equitable biomechanical competence as straight plates. The authors considered 45° helical plates as a valid alternative to straight plates from a biomechanical perspective; however, no dynamic cyclic testing was performed, and only artificial bones were used. The second biomechanical study compared 90° helical plates versus straight plates in a human cadaveric bone model under cyclic loading.^18^ The authors concluded that 90° helical plating was associated with lower resistance to flexion/extension and internal rotation with bigger shear interfragmentary displacements as compared to straight plating and therefore cannot be considered as its real alternative. In contrast, the results of the current study revealed no significant differences in resistance to torsional and shear displacement for 45° helical plates versus straight plates. This biomechanical behavior can be explained by the custom bending of both helical plate designs (45° and 90°) from straight plates, the possible weakening of their metallurgic structure and therefore by their impaired resistance against cyclic loading. Theoretically this effect is much bigger for 90° helical plates versus 45° helical plates because greater bending must be performed of the former, which is also reflected in the results of the current study. However, an industrially produced helical plate might behave differently as extensive bending procedures would be no longer necessary.

The main difference between 45° helical plates and straight plates in the current work is a trend to bigger axial stiffness of helical plates resulting in significant lower axial displacement and varus/valgus displacement compared to straight plates under axial load. This might be explained by the slightly bigger cross‐sectional area of the 45° helical plates resisting axial deformation—compared to straight plates when axially loaded. Interestingly, no further considerable differences were found between the investigated plate designs during both non‐destructive and destructive cyclic tests, rendering the 45° helical plate as a true alternative to straight plates in the treatment of proximal humeral shaft fractures. As a result, due to the anatomical advantages of their design, the 45° helical plate should be preferred over both straight and 90° helical plates during MIPO and ORIF. However, further research is needed to compare the biomechanical competence of the 45° helical plate design to ALPS during ORIF of proximal third humeral shaft fractures. The fact that the anterior strong part of the deltoid insertion must be released when ALPS is used renders the 45° helical plate as favorable during MIPO.

Several limitations of the current study must be considered. First, a cadaveric bone model is not capable to fully replicate the in vivo conditions after a fracture in a real human. Second, the major issue with biomechanical studies on the upper extremity is that it remains unclear which loading thresholds fixation constructs have to withstand in vivo.^39^ Up to now, there are no data in the literature on how many cycles the constructs must withstand for replication of uneventful bone healing. New technologies like continuous implant load monitoring to assess the bone healing status might bring new insights to this problem.^40^ Third, the plates used in the current study were custom‐bent into a 45° helical shape; therefore, their mechanical properties might have been impaired. An industrially produced implant might behave differently. However, all plates were bend in a standardized fashion avoiding weaking of the material to extensive bending maneuvers by one experienced trauma surgeon. Furthermore, cyclic loading was only performed in torsion, and different resistance to failure or different failure modes might be observed if the specimens are loaded axially. However, the upper extremities are predominantly loaded in torsion, which was the reason for choosing this loading protocol.^24^, ^25^

The strengths of the current study lie especially in the use of a precise motion tracking system. Furthermore, the use of paired cadaveric specimens allowed for a reliable assignment to the two study groups supported by a thorough BMD analysis of all used specimens. This biomechanical investigation adds valuable knowledge to the existing literature regarding the groundwork of the helical plating of the proximal humeral shaft. Future research should focus on the amount of anchorage loss in the humeral head when MIPO is applied, and not all proximal screws are occupied, as done in the current study. The observed anterior screw cut out in the humeral head might be explained with this. Future research could evaluate the use of bone cement augmentation in the humeral head to increase the screw anchorage especially when MIPO technique is used. Moreover, a biomechanical comparison between an industrially made 45° helical plate and the ALPS would be helpful. However, more clinical data are needed to evaluate the benefit of the helical plating technique as the number of clinical cases reported in the current literature is still low and other anatomical regions such as the thigh are shifting into focus of helical plating too.^41^, ^42^, ^43^

## 5 CONCLUSIONS

From a biomechanical perspective, 45° helical plating is associated with lower axial and varus/valgus displacement under axial loading and demonstrates comparable torsional and shear displacement under cyclic loading in internal rotation with similar resistance to failure versus straight plating. Therefore, 45° helical plates can be considered as a valid alternative to straight plates in the treatment of proximal humeral shaft fractures.

## AUTHOR CONTRIBUTIONS

Tatjana Pastor, Torsten Pastor, Boyko Gueorguiev, Ivan Zderic, and Frank J. P. Beeres designed the study. Tatjana Pastor and Ivan Zderic performed the preparation of the specimens and implanted them under supervision of Torsten Pastor. Tatjana Pastor and Ivan Zderic performed biomechanical testing. Ivan Zderic obtained data from optical motion tracking. Boyko Gueorguiev and Ivan Zderic performed statistical analysis. Boyko Gueorguiev, Ivan Zderic, TaB, Frank J. P. Beeres, Hristo Kostov Skulev, Ludmil Drenchev, and Tatjana Pastor interpreted results. R. Geoff Richards and Boyko Gueorguiev supervised the study. Tatjana Pastor wrote the original draft of the manuscript, revised in detail by Ivan Zderic, Nader Helmy, Frank J. P. Beeres, Philipp Kriechling, Boyko Gueorguiev, and Torsten Pastor. Subsequent drafts were prepared by all authors. All authors read and approved the final manuscript version.

## CONFLICT OF INTEREST STATEMENT

The authors declare no conflict of interest.

## ETHICS STATEMENT

All procedures performed in this study were followed in accordance with relevant guidelines. This study was approved by the institutional internal review board, based on the approval of the specimens’ delivery by Science Care Ethics Committee. All donors gave their informed consent inherent within the donation of the anatomical gift statement during their lifetime, as registered by Science Care.

## Data Availability

The datasets used and/or analyzed during the current study are available from the corresponding author on reasonable request.
